# Acute Thermal Tolerance and Physiological Responses in Commercial and Native Red-Feathered Roosters Sharing the Same HSP70 Homozygous Genotype

**DOI:** 10.3390/ani16121924

**Published:** 2026-06-22

**Authors:** Hsiao-Mei Liang, Der-Yuh Lin, Yan-Der Hsuuw, Kuo-Hsiang Hung

**Affiliations:** 1 Southern Region Branch, Taiwan Livestock Research Institute, Ministry of Agriculture, Pingtung 946, Taiwan; 2 Genetics and Physiology Division, Taiwan Livestock Research Institute, Ministry of Agriculture, Tainan 712, Taiwan; lin0429@tlri.gov.tw; 3 Department of Biological Science and Technology, National Pingtung University of Science and Technology, Pingtung 912, Taiwan; hsuuw@mail.npust.edu.tw; 4 Graduate Institute of Bioresources, National Pingtung University of Science and Technology, Pingtung 912, Taiwan; khhung424@mail.npust.edu.tw

**Keywords:** acute heat tolerance, breeding objective, HSP70 genotype, native chicken

## Abstract

Taiwan’s subtropical climate poses substantial heat stress challenges to poultry production. This study compared four red-feathered rooster lines (*n* = 10 per line, BB homozygous HSP70 genotype)—three commercially bred lines (F, T, K) selected for maximum body weight, and one native trial line (TLRI-09) developed through marker-assisted selection targeting the HSP70 BB genotype—during a one-hour acute heat challenge at 42 °C. A pre-specified statistical decision tree was applied: normality was assessed by the Shapiro–Wilk test for each group’s change score (Δ = post − pre); one-way ANOVA with Tukey’s HSD was used when all groups were normally distributed; Kruskal–Wallis with Dunn’s post hoc test (Bonferroni correction) was used otherwise. Within-group pre-to-post changes were assessed by paired *t*-test. TLRI-09 showed a substantially lower body weight (909 ± 102 g vs. 2039–2226 g) and zero mortality, whereas each commercial line experienced one death (10%). Cloacal temperatures in F, T, and K groups exceeded the thermometer’s upper limit (>44 °C) within one hour; TLRI-09 reached only 42.8 ± 0.1 °C. Respiratory rate increment was highest in TLRI-09 (Δ = 82.0 ± 8.4 breaths/min) and differed significantly among lines (*p* < 0.001). Plasma T_3_ change differed among lines (*p* = 0.006); post hoc analysis identified a significant K vs. T contrast only (*p* = 0.019). These results indicate that, despite sharing the same HSP70 genotype, breeding objective is an important determinant of acute thermal resilience—an observation that warrants further validation under chronic and commercial production conditions.

## Simple Summary

This study examined whether chickens sharing the same heat-related gene (HSP70 BB genotype) differ in acute heat tolerance when their breeding objectives diverge. Four red-feathered rooster lines were compared: three commercial lines selected for high body weight at market age (12–16 weeks), and one native line (TLRI-09) developed through marker-assisted selection targeting the HSP70 BB genotype. All lines were subjected to a standardized one-hour acute heat challenge at 42 °C. Although all lines shared the same HSP70 genotype, TLRI-09 showed more favorable physiological responses under acute heat stress, including a lower increase in cloacal temperature, a higher respiratory rate response, and no mortality, compared with 10% mortality and cloacal temperatures exceeding the measurable limit (>44 °C) in each commercial line. These results suggest that differences in breeding objectives may contribute to variation in acute heat tolerance beyond HSP70 genotype alone, with potential relevance for understanding heat stress responses in poultry under acute heat stress in subtropical production conditions.

## 1. Introduction

Taiwan is situated in the subtropical zone, where mean summer temperatures have ranged from 28 to 29 °C in recent years [1]. Heat stress is a primary environmental stressor in poultry production in Taiwan, causing reductions in feed intake, growth rate, egg production, and immune function, as well as elevated mortality—particularly during the transport of live birds to slaughterhouses using open trucks without temperature control. Journeys lasting one to two hours in summer conditions expose birds to potentially lethal thermal loads, and transport-related mortality represents a significant welfare and economic concern for Taiwan’s native chicken industry.

Heat tolerance in chickens is a complex, polygenic trait influenced by multiple physiological and molecular mechanisms. These include thermoregulatory responses mediated by the hypothalamic–pituitary–adrenal (HPA) and hypothalamic–pituitary–thyroid (HPT) axes [2], cardiovascular adaptations such as increased cardiac output and peripheral vasodilation [3], antioxidant defense systems that mitigate reactive oxygen species generated during heat stress [4], and other members of the heat shock protein family (e.g., HSP70, HSP90) that contribute to cellular protein homeostasis under thermal challenge [5]. Epigenetic modifications and breed-specific differences in feather coverage and body composition also modulate overall heat tolerance [6,7]. Selecting thermotolerant chicken lines is therefore an effective but multifactorial strategy for mitigating heat stress in hot climates [8].

The heat shock protein 70 (HSP70) gene is a key molecular marker associated with thermotolerance in poultry [9]. Studies suggest that intensive phenotypic selection for rapid growth rate in commercial broiler lines has progressively narrowed their thermal safety margins, increasing susceptibility to heat-related mortality [10]. In contrast, native or slow-growing breeds with less intensive selection histories tend to exhibit greater heat adaptation [11]. The HSP70 genotype has been identified as a useful selection marker: Liang et al. (2016) demonstrated that, within a single line, birds carrying the homozygous genotype exhibited superior acute heat tolerance and maintained growth performance and egg production compared to heterozygous genotype birds [12]. Duangjinda et al. (2017) similarly reported that the C_1_C_1_ genotype conferred heat-tolerance advantages across commercial and native chickens reared under hot and humid conditions, while the C_2_C_2_ genotype (homozygous for the alternative allele) was associated with lower heat tolerance [13].

Within the red-feathered chicken industry in Taiwan, commercial breeding farms have independently maintained closed parent stock populations using phenotypic mass selection, with body weight as the dominant selection criterion—reflecting Taiwan’s practice of pricing live poultry by weight. The Taiwan Livestock Research Institute (MOA-TLRI) subsequently developed TLRI-09, an experimental native line, through marker-assisted selection using the HSP70 BB genotype as a primary criterion, with red-feathered native Taiwanese chickens as the base population. The design rationale builds directly on Liang et al. (2016): having established that HSP70 genotype polymorphisms significantly influence heat tolerance within a single line [12], the present study asks whether the same HSP70 BB genotype produces different acute thermal outcomes across lines with divergent breeding objectives. By restricting all experimental birds to the BB genotype, we isolated the effect of breeding objective from HSP70 genotype variation.

The objectives of this study were therefore: (1) to compare acute physiological responses to a standardized heat challenge (42 °C, 1 h) among four red-feathered rooster lines sharing the same HSP70 BB genotype; and (2) to evaluate whether breeding objective—specifically, phenotypic mass selection for maximum body weight at market age (12–16 weeks) versus HSP70-targeted marker-assisted selection—explains between-line differences in acute thermal resilience.

## 2. Materials and Methods

### 2.1. Experimental Animals and Breeding Background

A total of 392 day-old male chicks from three commercial breeding farms in Taiwan (F, T, and K groups) and 109 day-old male chicks from the native chicken line maintained at MOA-TLRI (TLRI-09) were reared under identical conditions in the same poultry house, with each group allocated to separate floor pens. All groups were managed by the same personnel following identical husbandry protocols (feed, water, lighting, ventilation, and ambient temperature control) from day-old to 12 weeks. Male birds were used exclusively to avoid confounding by the reproductive cycle, ovarian hormonal fluctuations, and the physiological demands of egg production, which can substantially alter thermoregulatory physiology and baseline T_3_ levels in females; use of roosters provides a standardized physiological baseline for between-line comparisons. Future studies should include female birds to assess sex-specific heat stress responses.

Although maintained as independent closed flocks by separate private farms, the F, T, and K commercial lines shared an identical breeding objective: maximizing body weight at market age, specifically targeting the heaviest possible live weight between 12 and 16 weeks of age—reflecting Taiwan’s commercial practice of pricing live poultry by body weight at slaughter. In all three lines, the heaviest individuals were preferentially retained as breeding stock across successive generations, with no explicit selection pressure applied to thermotolerance or molecular markers. TLRI-09 was developed by MOA-TLRI through marker-assisted selection using the HSP70 BB homozygous genotype as a primary selection criterion alongside productive performance targets, with red-feathered native Taiwanese chickens as the base population.

All animal experimental procedures were approved by the Institutional Animal Care and Use Committee (IACUC) of the Kaohsiung Animal Propagation Station, Taiwan Livestock Research Institute (Approval No. KAPS-LRI#107-5) and performed in accordance with applicable guidelines.

### 2.2. HSP70 Genotyping

At 12 weeks of age, 470 roosters were genotyped by PCR–SSCP analysis following Mazzi et al. (2003) [14], identifying a SNP at position 258 of the HSP70 gene. Genotypes were classified as AA, AB, or BB. TLRI-09 consisted entirely of BB homozygous individuals (109/109; 100%). From each of the commercial lines, BB homozygous birds were identified and selected for the acute heat tolerance test.

### 2.3. Acute Heat Tolerance Test

From each line, 10 roosters carrying the BB homozygous genotype were selected for the acute heat tolerance test (*n* = 5 per group per replicate; two replicates conducted one week apart). Birds were selected based on proximity to the group mean body weight at 12 weeks of age, with five individuals per replicate chosen from above and below the mean to ensure representative sampling. Both replicates were conducted during the same season (May–June), in the same climate-controlled chamber, managed by the same personnel, ensuring environmental consistency. Statistical confirmation of negligible replicate effects is provided in Section 2.5.

The heat challenge was performed in an environmental chamber maintained at 42 °C and 55–65% relative humidity for one hour, preceded by a 30 min acclimation period at 25 °C. The 42 °C challenge temperature was selected because: (1) the lethal threshold for adult chickens is approximately 46 °C [12]; (2) 42 °C was expected to induce measurable physiological responses without widespread mortality; and (3) it approximates the thermal conditions that may be experienced during open-truck transport of live birds to slaughterhouses in Taiwan during summer, with transit times up to two hours. The original protocol planned a 2 h exposure; however, this was shortened to 1 h following mortality events in commercial group birds during the first replicate trial.

### 2.4. Measurements

Respiratory rate (breaths per minute) was determined by counting visible abdominal movements for one minute at 0 h (immediately before heat exposure) and 1 h (at the end of the heat challenge). Cloacal temperature was measured using a digital thermometer (K-Jump Health Co., Ltd., New Taipei City, Taiwan; measurement range: 32–44 °C). Based on the normal cloacal temperature range of adult chickens (40.5–41.7 °C) [15,16,17], the thermometer range was considered sufficient a priori; however, cloacal temperatures in the commercial groups exceeded the upper instrument limit (>44 °C) during heat exposure, and thus exact 1 h values for these groups could not be recorded. Accordingly, 1 h cloacal temperature for the F, T, and K groups is reported descriptively as >44 °C, and no statistical comparison of cloacal temperature at 1 h among commercial lines was performed. Future studies should use thermometers with a measurement range of at least 46 °C. Blood samples (2 mL) were collected via wing vein puncture at 0 h and 1 h for measurement of plasma T_3_ concentration (ng/mL) by radioimmunoassay.

### 2.5. Statistical Analysis

Data are presented as mean ± SEM. Data were analyzed using SPSS Statistics version 19.0 (IBM Corp., Armonk, NY, USA). A pre-specified statistical decision tree was applied to determine the appropriate between-line test for each endpoint. (1) Normality of each group’s change score (Δ = post − pre) was assessed by the Shapiro–Wilk test. (2) If all four groups passed normality, one-way ANOVA with Tukey’s Honest Significant Difference (HSD) post hoc test was applied. (3) If one or more groups failed normality (*p* < 0.05), the Kruskal–Wallis test was used, with Dunn’s post hoc test and Bonferroni correction for pairwise comparisons. (4) Within-group pre-to-post changes were assessed by paired *t*-test for each line. (5) Body weight differences across lines and age points were analyzed by one-way ANOVA with Tukey’s HSD.

Replicate effects were assessed prior to pooling: independent-samples *t*-tests comparing Replicate 1 vs. Replicate 2 within each line showed no significant differences for any physiological endpoint (all *p* > 0.13; overall replicate effect *p* = 0.872). Variance homogeneity across lines was confirmed by Bartlett’s test (respiratory rate increment: χ^2^ = 6.50, *p* = 0.090) and Levene’s test (F = 1.01, *p* = 0.401), supporting the use of one-way ANOVA for normally distributed endpoints. Because line and 12-week body weight are inherently collinear (reflecting cumulative divergent selection histories), body weight was not included as a covariate in the primary analysis; however, a supplementary ANCOVA is reported in Section 4.2.

Mortality rate comparison (1 death per commercial group vs. 0 in TLRI-09) was assessed by Fisher’s exact test; however, given *n* = 10 per group, this comparison is presented as descriptive only and should be interpreted with caution.

## 3. Results

### 3.1. Distribution of HSP70 Genotypes

As shown in Table 1, TLRI-09 consisted entirely of BB homozygous individuals (109/109; 100%), reflecting sustained selection targeting the HSP70 BB genotype. Among the commercial lines, BB frequency was approximately 24–27% (F: 31/128, 24.2%; T: 30/112, 26.8%; K: 32/121, 26.4%), consistent with Hardy–Weinberg expectations for populations not under direct selection at this locus.

### 3.2. Body Weight at Different Weeks of Age

Body weights are presented in Table 2. At birth (0 wk), TLRI-09 chicks were significantly lighter than commercial groups (30 ± 0.5 g vs. 35–38 g; *p* < 0.05). The weight divergence increased markedly over time. At 12 weeks, T (2226 ± 121 g) and K (2141 ± 132 g) did not differ significantly from each other but were significantly heavier than F (2039 ± 113 g), which in turn was significantly heavier than TLRI-09 (909 ± 102 g; all *p* < 0.05, Tukey’s HSD). The 12-week body weight of TLRI-09 was approximately 41–45% of the commercial line weights, reflecting the divergent breeding objectives.

### 3.3. Physiological Responses to Acute Heat Stress

Respiratory rate and plasma T_3_ results are presented in Table 3. Cloacal temperature and mortality outcomes are described below.

#### 3.3.1. Cloacal Temperature

Baseline cloacal temperatures were within the normal range for adult chickens across all lines (41.6–41.9 °C) and did not differ significantly among groups. After one hour at 42 °C, cloacal temperatures in the F, T, and K groups all exceeded the thermometer’s upper measurement limit (>44 °C), precluding exact recording and statistical comparison. The minimum temperature increment for these commercial groups was therefore ≥2.1–2.3 °C from baseline. In contrast, TLRI-09 reached a mean cloacal temperature of 42.8 ± 0.1 °C at 1 h, representing an increment of only 1.2 ± 0.1 °C—substantially lower than the minimum estimated increments in commercial groups. The thermometer limitation is acknowledged as a methodological constraint; future studies should employ instruments with a range of at least 46 °C.

#### 3.3.2. Mortality

One bird died in each of the F, T, and K groups during the one-hour heat challenge (10% per group); no mortality occurred in TLRI-09 (0%). Given the small sample size (*n* = 10 per group), formal statistical comparison of mortality rates is not appropriate, and these figures are reported descriptively only. All deaths occurred during the heat exposure period and were temporally associated with extreme hyperthermia (cloacal temperature > 44 °C), consistent with heat-related mortality.

#### 3.3.3. Respiratory Rate

Pre-exposure respiratory rates did not differ significantly among lines (43.3–58.7 breaths/min). After heat exposure, all groups showed a significant increase from baseline (paired *t*-test: all *p* ≤ 0.01). The respiratory rate increment (Δ) differed significantly among lines (one-way ANOVA: F = 11.49, *p* < 0.001; Shapiro–Wilk normality confirmed for all four groups). Tukey’s HSD post hoc analysis revealed that TLRI-09 (Δ = 82.0 ± 8.4 breaths/min) had a significantly greater increment than all three commercial lines (all *p* < 0.001). The F group (Δ = 41.3 ± 16.3) and K group (Δ = 44.4 ± 14.4) did not differ significantly from each other but were significantly higher than the T group (Δ = 12.6 ± 10.6; *p* < 0.05).

#### 3.3.4. Plasma T_3_ Concentration

Pre-exposure plasma T_3_ concentrations did not differ significantly among lines (36.6–56.9 ng/mL). Following heat exposure, T_3_ decreased significantly within the F group (paired *t*-test: *p* < 0.05) and T group (*p* < 0.001), but not in the K or TLRI-09 groups. The between-line comparison of T_3_ change scores (Δ) was significant (Kruskal–Wallis: H = 12.39, df = 3, *p* = 0.006; Shapiro–Wilk test indicated non-normality in at least one group, justifying the non-parametric approach). Dunn’s post hoc test with Bonferroni correction identified a significant difference between the K and T groups only (adjusted *p* = 0.019); no other pairwise comparisons reached significance after correction.

## 4. Discussion

### 4.1. Contrasting Breeding Strategies Shape Thermal Resilience Beyond HSP70 Genotype

The central finding of this study is that four red-feathered rooster lines sharing the identical HSP70 BB homozygous genotype exhibited markedly different physiological responses to a standardized acute heat challenge. TLRI-09—developed through HSP70-targeted marker-assisted selection—demonstrated substantially superior acute heat tolerance compared with all three commercially bred lines, as reflected by a lower cloacal temperature rise, a higher respiratory rate increment, and zero mortality. This outcome demonstrates that the HSP70 BB genotype alone does not guarantee equivalent thermal tolerance across lines with different breeding histories.

The commercial F, T, and K lines were established through phenotypic mass selection sharing an identical objective: maximizing body weight at market age (12–16 weeks), an approach analogous to the intensive directional selection for rapid growth applied in global commercial broiler breeding. All three lines applied the same selection criterion—retaining the heaviest individuals as breeding stock each generation—independent of any thermotolerance measure. Studies suggest that this type of weight-focused selection progressively narrows thermal safety margins, and slow-growing chickens were more resilient to heat stress than faster-growing genotypes [11,18]. The HSP70 locus may also interact with other genomic regions through linkage disequilibrium or epistatic effects, and controlling a single SNP does not completely eliminate genomic background differences among lines [19]. The present results are consistent with previous studies that demonstrated that native and slow-growing lines with less intensive selection histories retain greater thermal resilience than fast-growing commercial counterparts [11,18].

The updated statistical framework—employing a pre-specified decision tree with Shapiro–Wilk normality testing followed by one-way ANOVA/Tukey’s HSD for respiratory rate (where normality held) and Kruskal–Wallis/Dunn’s test for plasma T_3_ (where normality failed in at least one group)—provides a more rigorous basis for between-line inference than the LSD-based analysis used previously. The consistent detection of TLRI-09′s superior respiratory rate increment (Tukey’s HSD: all *p* < 0.001) across this framework strengthens confidence in the between-line physiological differences.

Collectively, these findings support the interpretation that divergent breeding strategies—particularly long-term selection for rapid growth versus HSP70-targeted marker-assisted selection—substantially shape acute thermal resilience beyond the effect of HSP70 genotype alone.

### 4.2. Body Size, Metabolic Heat Production, and Thermal Vulnerability

The substantial difference in body weight between TLRI-09 and the commercial lines (Table 2) is directly relevant to thermal vulnerability. Larger, fast-growing birds generate more metabolic heat per unit time and have a less favorable surface-area-to-body-mass ratio for radiative and convective heat dissipation [20,21]. Fast-growing commercial broilers have been widely reported to exhibit greater susceptibility to heat stress due to elevated metabolic heat production and reduced heat dissipation efficiency compared with lighter or slower-growing lines [22]. This creates a physiological disadvantage under acute heat stress that compounds with increased age and weight.

To address the inherent confounding among breeding objectives, body weight, growth trajectory, and metabolic rate, we conducted a supplementary ANCOVA incorporating 12-week body weight as a covariate. Body weight was negatively correlated with respiratory rate increment (Pearson r = −0.547, *p* < 0.001), confirming that heavier birds tended to show smaller panting responses. After adjusting for body weight, the line effect on respiratory rate increment was attenuated (ANCOVA: F = 2.38, *p* = 0.107), reflecting the high collinearity between line and body weight, as expected given their shared origin in divergent selection histories. Adjusted means remained highest in TLRI-09 (78.3 breaths/min) and lowest in the T group (13.2 breaths/min). These results confirm that body weight partially contributes to observed between-line variation; however, the source of those weight differences is itself a breeding objective. Previous reviews have similarly emphasized that genetic selection for rapid growth and meat yield can inadvertently reduce thermotolerance and increase vulnerability to heat stress in poultry [22,23]. Future studies employing weight-matched comparisons will be needed to further disentangle these effects.

### 4.3. Respiratory Rate: Effective Panting Versus Thermoregulatory Failure

TLRI-09 exhibited the highest respiratory rate increment (Δ = 82.0 ± 8.4 breaths/min; Tukey’s HSD vs. all commercial lines: all *p* < 0.001) yet the lowest cloacal temperature rise and zero mortality. This dissociation indicates that elevated respiratory activity in TLRI-09 was associated with effective evaporative heat dissipation rather than systemic thermoregulatory failure. In poultry, panting is the primary mechanism for latent heat loss under heat stress; however, its effectiveness depends on whether respiratory compensation is sufficient to maintain core thermal homeostasis [22,24,25]. Accordingly, respiratory rate should be interpreted in combination with cloacal temperature and survival outcomes rather than as a standalone indicator of heat stress severity. Conversely, the lower respiratory rate increment observed in commercial lines—particularly the T group (Δ = 12.6 ± 10.6 breaths/min)—coincided with cloacal temperatures exceeding measurable limits and a 10% mortality rate, consistent with severe thermoregulatory failure. Under extreme heat stress, failure of evaporative cooling capacity may lead to progressive hyperthermia, physiological decompensation, and mortality [24,26]. In highly selected broiler lines, reduced heat tolerance has been widely associated with an imbalance between metabolic heat production and dissipative capacity, increasing susceptibility to heat-induced collapse [27]. An important limitation of this study is the use of a single post-exposure measurement time point, which prevents assessment of the temporal trajectory of respiratory responses. Therefore, whether the low respiratory increment in commercial lines reflects insufficient compensatory response or a late-stage physiological decline cannot be determined. From a stress physiology perspective, extreme environmental stress may overwhelm regulatory capacity, leading to functional decompensation rather than adaptive adjustment [28]. Accordingly, the present interpretation remains plausible but requires validation through repeated time-course measurements and blood gas/acid–base analysis.

### 4.4. Plasma T_3_ and the Thyroid Axis Under Acute Heat Exposure

The between-line comparison of plasma T_3_ Δ was significant (Kruskal–Wallis: *p* = 0.006), yet the only significant post hoc contrast after Bonferroni correction was K vs. T (Dunn’s test: adjusted *p* = 0.019). This limited pattern of significance may reflect the small sample size (*n* = 9–10 per group), the large inter-individual variation in TLRI-09 (a characteristic of native breeds with less intensive selection history), and the short exposure duration (one hour). High within-line variability in physiological and endocrine responses has been commonly reported in indigenous or less intensively selected poultry populations, particularly under acute heat stress conditions [23,24]. The significant T_3_ decrease in the F and T groups (paired *t*-test: *p* < 0.05 and *p* < 0.001, respectively) is consistent with previous reports describing suppression of thyroid activity under acute heat stress, which is interpreted as an adaptive mechanism to reduce metabolic heat production [26,28]. Heat-induced reductions in circulating thyroid hormones have been associated with decreased metabolic rate and improved short-term thermal balance in poultry exposed to elevated environmental temperatures [23]. The absence of significant T_3_ change in K and TLRI-09 groups under the current experimental conditions represents a null result for this endpoint and should not be interpreted as evidence against a thyroid axis response under more prolonged or severe thermal challenges. Thyroid-related metabolic adjustments are known to be time- and intensity-dependent, and may become more pronounced under sustained heat exposure or higher thermal load conditions [24,26].

### 4.5. Cloacal Temperature and Mortality as Clinical Indicators

Cloacal temperature proved to be the most discriminating variable in this study. All three commercial lines exceeded 44 °C within one hour—the zone associated with severe hyperthermia and approaching the upper physiological limit of thermoregulation in poultry—while TLRI-09 stabilized at 42.8 °C. Core body temperature is widely recognized as the most reliable indicator of heat stress severity in poultry, as peripheral indicators such as respiratory rate may reflect compensatory responses rather than true physiological failure [24,26]. The inability to precisely quantify temperatures above 44 °C due to instrument limitations represents a methodological constraint, as accurate assessment of extreme hyperthermia requires measurement ranges extending beyond typical physiological thresholds in broilers under acute heat stress conditions. Mortality was confined to commercial lines (one bird per group, 10%). Although the small sample size precludes statistical inference on mortality, the temporal association with severe hyperthermia is consistent with previous reports indicating that elevated core body temperature is strongly associated with physiological collapse and increased mortality risk in heat-stressed poultry [23,26]. The zero mortality observed in TLRI-09 is consistent with its lower cloacal temperature and more effective thermoregulatory response, as maintenance of core temperature within tolerable limits is a key determinant of survival under acute heat stress.

### 4.6. Implications for Native Chicken Breeding Programs

The results demonstrate that the HSP70 BB genotype alone does not guarantee equivalent thermal tolerance across lines with different selection histories. Genetic resistance to heat stress is a complex quantitative trait influenced by multiple interacting physiological and metabolic pathways, and single candidate gene selection is unlikely to fully explain phenotypic variation under heat stress conditions [23,26]. TLRI-09’s superior acute heat tolerance likely reflects the combined effects of: (1) HSP70-targeted selection, which may have contributed to improved cellular stress response capacity; (2) substantially lower body weight and associated metabolic heat production, a key determinant of heat dissipation efficiency; and (3) additional unmeasured differences in genetic background, body composition, and basal metabolic rate [22,24]. Disentangling these contributions requires future studies with weight-matched experimental designs, molecular-level endpoints (including HSP70 protein expression and oxidative stress biomarkers), and evaluation under both acute and chronic heat stress conditions. Heat stress responses in poultry are highly dependent on exposure duration and intensity, and chronic heat exposure may elicit different endocrine and metabolic adaptations compared with acute challenges [23,29]. Furthermore, inclusion of female birds is necessary to assess sex-specific responses, particularly given the well-documented sensitivity of ovarian function and egg production to elevated environmental temperature [26]. It should be noted that the present study employed an acute heat challenge model (42 °C, 1 h), which may not fully replicate the chronic, lower-intensity heat stress conditions typical of subtropical poultry production systems. However, this model is relevant to practical production scenarios in Taiwan, as it can mimic the short-duration but severe thermal exposure experienced during live-bird transportation from farms to slaughterhouses, typically lasting approximately 1–2 h. Therefore, the present findings provide meaningful insights into physiological responses and tolerance differences under acute thermal shock conditions. Nevertheless, extrapolation of these findings to field conditions should be made cautiously, and validation under commercial production environments is required before implementation in breeding programs. Nevertheless, the findings suggest that incorporating HSP70-associated selection into native chicken breeding programs may represent a promising strategy to enhance thermal resilience in subtropical poultry systems. However, given the multifactorial nature of heat tolerance, such approaches should be integrated with selection for metabolic efficiency and growth traits to achieve balanced genetic improvement under increasing climatic stress [23,24].

## 5. Conclusions

Under the specific conditions of this study (40 roosters, 12 weeks of age, exposed to 42 °C for one hour), the native TLRI-09 line—developed through marker-assisted selection targeting the HSP70 BB genotype—showed higher acute heat tolerance compared with three commercially bred lines sharing the same HSP70 BB genotype. Although all lines were fixed for the HSP70 BB genotype prior to analysis, differences in acute thermophysiological responses were still observed among lines. These results suggest that the HSP70 genotype alone may not be sufficient as a single marker to fully explain variation in thermotolerance, and that additional physiological and polygenic factors are likely involved in acute heat responses. Differences in breeding objectives—particularly between selection for high body weight and marker-assisted selection targeting HSP70—may also contribute to variation in thermal resilience beyond genotype alone. However, causal interpretation is limited by confounding among line, body weight, and genetic background. Further studies under chronic heat stress and commercial production conditions are required to better evaluate the robustness and practical relevance of these findings before any broader application in breeding programs.

## Figures and Tables

**Table 1:** HSP70 genotype distribution in the four experimental rooster lines.

Group	AA	AB	BB	Total
F Group	33	64	31	128
T Group	27	55	30	112
K Group	29	60	32	121
TLRI-09	0	0	109	109
Total	89	179	202	470

Values represent number of birds.

**Table 2:** Body weight (g) of the four experimental rooster lines at different weeks of age.

Week of Age	F Group	T Group	K Group	TLRI-09
*n*	128	112	121	109
0 wk	38 ± 0.8 ^a^	37 ± 0.7 ^a^	35 ± 1.2 ^ab^	30 ± 0.5 ^b^
4 wk	438 ± 32 ^b^	492 ± 23 ^a^	452 ± 35 ^b^	223 ± 29 ^c^
8 wk	1335 ± 92 ^c^	1632 ± 125 ^a^	1517± 84 ^b^	525 ± 75 ^d^
12 wk	2039 ± 113 ^b^	2226 ± 121 ^a^	2141 ± 132 ^ab^	909 ± 102 ^c^

Mean ± SEM. ^a,b,c,d^ Means in the same row with different superscripts differ significantly (*p* < 0.05; one-way ANOVA followed by Tukey’s HSD).

**Table 3:** Physiological responses of four rooster lines to acute heat stress at 42 °C for one hour.

Trait	F Group (*n* = 9)	T Group (*n* = 9)	K Group (*n* = 9)	TLRI-09 (*n* = 10)
Respiratory rate (breaths/min)				
0 h	58.7 ± 6.6	51.3 ± 2.6	46.3 ± 5.5	43.3 ± 5.5
1 h (42 °C)	98.3 ± 10.7	62.5 ± 2.5	90.7 ± 14.9	125.3 ± 6.2
Δ (increment)	41.3 ± 16.3 ^b^	12.6 ± 10.6 ^c^	44.4 ± 14.4 ^b^	82.0 ± 8.4 ^a^
Paired *t*-test (0 h vs. 1 h)	***	**	***	***
Between-line: one-way ANOVA, F = 11.49, *p* < 0.001; Tukey’s HSD: TLRI-09 > F = T = K (all *p* < 0.001)				
Plasma T_3_ concentration (ng/mL)				
0 h	47.8 ± 2.1	46.7 ± 7.2	36.6 ± 5.8	56.9 ± 17.4
1 h (42 °C)	39.4 ± 9.2	34.2 ± 7.9	36.4 ± 5.0	50.5 ± 20.8
Δ (change)	−8.0 ± 9.9	−11.3 ± 5.9	+0.7 ± 5.1	−6.4 ± 25.5
Paired *t*-test (0 h vs. 1 h)	*	***	ns	ns
Between-line: Kruskal–Wallis, H = 12.39, *p* = 0.006; Dunn’s post hoc (Bonferroni): K vs. T, adjusted *p* = 0.019; all other pairs ns				
Supplementary ANCOVA (covariate: 12-wk body weight)				
Adjusted RR increment (breaths/min) †	27.8	13.2	33.6	78.3
Line effect after BW adjustment: F = 2.38, *p* = 0.107 (ns); BW × RR increment: Pearson r = −0.547, *p* < 0.001				

Mean ± SEM. ^a,b,c^ Means in the same row with different superscripts differ significantly (*p* < 0.05; Tukey’s HSD, applied to respiratory rate increment only). Paired *t*-test: * *p* < 0.05; ** *p* < 0.01; *** *p* < 0.001; ns, not significant. Cloacal temperatures in F, T, and K exceeded the thermometer’s upper limit (>44 °C) at 1 h; data reported descriptively in Section 3.3.1 only. The larger SE for TLRI-09 T_3_ reflects greater inter-individual variation characteristic of native breeds with less intensive selection history and the small sample size (*n* = 10). † ANCOVA adjusted means estimated at the grand mean body weight (1579 g); line effect non-significant after body weight adjustment (*p* = 0.107), reflecting collinearity between line and 12-wk body weight (Pearson *r* = −0.547, *p* < 0.001).

## Data Availability

The original contributions presented in this study are included in the article. Further inquiries can be directed to the corresponding author.

## References

[1] Lau T.K., Hsu H.C. (2025). Urban heat island effect and unequal temperature-related news attention in Taiwan’s major cities. Urban Sci..

[2] Moberg G.P., Moberg G.P., Mench J.A. (2000). Biological response to stress: Implications for animal welfare. The Biology of Animal Stress: Basic Principles and Implications for Animal Welfare.

[3] Wang S., Bottje W.G., Kinzler S., Neldon H.L., Koike T.I. (1989). Effect of heat stress on plasma levels of arginine vasotocin and mesotocin in domestic fowl (Gallus domesticus). Comp. Biochem. Physiol. A Comp. Physiol..

[4] Nakamura K. (2011). Central circuitries for body temperature regulation and fever. Am. J. Physiol. Regul. Integr. Comp. Physiol..

[5] De Maio A. (1999). Heat shock proteins: Facts, thoughts, and dreams. Shock.

[6] Gaviol H.C., Gasparino E., Prioli J.A., Soares M.A. (2008). Genetic evaluation of the HSP70 protein in the Japanese quail (Coturnix japonica). Genet. Mol. Res..

[7] Yunis R., Cahaner A. (1999). The effects of the naked neck (Na) and frizzle (F) genes on growth and meat yield of broilers and their interactions with ambient temperatures and potential growth rate. Poult. Sci..

[8] Horst P. (1989). Native fowl as reservoir for genomes and major genes with direct and indirect effects on adaptability and their potential for tropically oriented breeding plans. Eur. Poult. Sci..

[9] Tamzil M.H., Noor R.R., Hardjosworo P.S., Manalu W., Sumantri C. (2013). Acute heat stress responses of three lines of chickens with different heat shock protein (HSP)-70 genotypes. Int. J. Poult. Sci..

[10] Lin H., Jiao H.C., Buyse J., Decuypere E. (2006). Strategies for preventing heat stress in poultry. World’s Poult. Sci. J..

[11] Soleimani A.F., Zulkifli I., Omar A.R., Raha A.R. (2011). Physiological responses of three chicken breeds to acute heat stress. Poult. Sci..

[12] Liang H.-M., Lin D.-Y., Hsuuw Y.-D., Huang T.-P., Chang H.-L., Lin C.-Y., Wu H.-H., Hung K.-H. (2016). Association of heat shock protein 70 gene polymorphisms with acute thermal tolerance, growth, and egg production traits of native chickens in Taiwan. Arch. Anim. Breed..

[13] Duangjinda M., Tunim S., Duangdaen C., Boonkum W. (2017). HSP70 genotypes and heat tolerance of commercial and native chickens reared in hot and humid conditions. Braz. J. Poult. Sci..

[14] Mazzi C.M., Ferro J.A., Ferro M.I.T., Savino V.J.M., Coelho A.A.D., Macari M. (2003). Polymorphism analysis of the HSP70 stress gene in broiler chickens (Gallus gallus) of different breeds. Genet. Mol. Biol..

[15] Aho P.W., Donald D.B., William D.W. (2002). Feed and the poultry industry. Commercial Chicken Meat and Egg Production.

[16] Daghir N.J. (2008). Poultry Production in Hot Climates.

[17] Damerow G. (2016). Mysteries of metabolism. The Chicken Health Handbook.

[18] Pontalti E., Pirrone F., Dalle Zotte A., Nalon E., Verdiglione R., Mattioli S., Birolo M. (2025). Impact of heat stress on growth performance and carcass traits of fast-, medium- and slow-growing broiler chicken genotypes. Poult. Sci..

[19] Wadood A.A., Rehman S.U., Bordbar F., Zhang X. (2026). Comparative genomic and evolutionary analysis of the heat shock protein gene family in the chicken genome. Anim. Biotechnol..

[20] Tickle P.G., Codd J.R. (2019). Thermoregulation in rapidly growing broiler chickens is compromised by constraints on radiative and convective cooling performance. J. Therm. Biol..

[21] Ruuskanen S., Hsu B.Y., Nord A. (2021). Endocrinology of thermoregulation in birds in a changing climate. Mol. Cell. Endocrinol..

[22] Brugaletta G., Teyssier J.R., Rochell S.J., Dridi S., Sirri F. (2022). A review of heat stress in chickens. Part I: Insights into physiology and gut health. Front. Physiol..

[23] Nawaz A.H., Amoah K., Leng Q.Y., Zheng J.H., Zhang W.L., Zhang L. (2021). Poultry response to heat stress: Physiological, metabolic, and genetic implications on meat production and quality. Front. Vet. Sci..

[24] Renaudeau D., Collin A., Yahav S., de Basilio V., Gourdine J.L. (2012). Adaptation to hot climate and strategies to alleviate heat stress in livestock production. Animal.

[25] Idowu P.A., Chauke C., Mpofu T.J. (2026). Integrated stress physiology and mitigation strategies for heat stress in layer chickens. Animals.

[26] Lara L.J., Rostagno M.H. (2013). Impact of heat stress on poultry production. Animals.

[27] Oluwagbenga E.M., Fraley G.S. (2023). Heat stress and poultry production: A comprehensive review. Poult. Sci..

[28] Yahav S. (2009). Alleviating heat stress in domestic fowl: Different strategies. World’s Poult. Sci. J..

[29] Wasti S., Sah N., Mishra B. (2020). Impact of heat stress on poultry health and performance, and mitigation strategies. Animals.

